# Generation and Characterization of Multipotent Stem Cells from Established Dermal Cultures

**DOI:** 10.1371/journal.pone.0050742

**Published:** 2012-11-30

**Authors:** Rebecca P. Hill, Karl Gledhill, Aaron Gardner, Claire A. Higgins, Heather Crawford, Clifford Lawrence, Christopher J. Hutchison, William A. Owens, Bo Kara, S. Elizabeth James, Colin A. B. Jahoda

**Affiliations:** 1 School of Biological and Biomedical Sciences, Durham University, Durham, County Durham, United Kingdom; 2 Fujifilm Diosynth, Billingham, Cleveland, United Kingdom; 3 Department of Dermatology, Columbia University, New York, New York, United States of America; 4 Department of Cardiothoracic Surgery, James Cook University Hospital, Middlesbrough, Cleveland, United Kingdom; 5 Department of Dermatology, Royal Victoria Infirmary, Newcastle-upon-Tyne, United Kingdom; 6 School of Pharmacy and Biomolecular Sciences, University of Brighton, Brighton, East Sussex, United Kingdom; University of Newcastle upon Tyne, United Kingdom

## Abstract

Human multipotent skin derived precursor cells (SKPs) are traditionally sourced from dissociated dermal tissues; therefore, donor availability may become limiting. Here we demonstrate that both normal and diseased adult human dermal fibroblasts (DF) pre-cultured in conventional monolayers are capable of forming SKPs (termed m-SKPs). Moreover, we show that these m-SKPs can be passaged and that cryopreservation of original fibroblast monolayer cultures does not reduce m-SKP yield; however, extensive monolayer passaging does. Like SKPs generated from dissociated dermis, these m-SKPs expressed nestin, fibronectin and versican at the protein level. At the transcriptional level, m-SKPs derived from normal adult human DF, expressed neural crest stem cell markers such as *p75NTR*, embryonic stem cell markers such as *Nanog* and the mesenchymal stem cell marker *Dermo-1*. Furthermore, appropriate stimuli induced m-SKPs to differentiate down either mesenchymal or neural lineages resulting in lipid accumulation, calcification and S100β or β-III tubulin expression (with multiple processes). m-SKP yield was greater from neonatal foreskin cultures compared to those from adult DF cultures; however, the former showed a greater decrease in m-SKP forming capacity after extensive monolayer passaging. m-SKP yield was greater from adult DF cultures expressing more alpha-smooth muscle actin (αSMA). In turn, elevated αSMA expression correlated with cells originating from specimens isolated from biopsies containing more terminal hair follicles; however, αSMA expression was lost upon m-SKP formation. Others have shown that dissociated human hair follicle dermal papilla (DP) are a highly enriched source of SKPs. However, conversely and unexpectedly, monolayer cultured human hair follicle DP cells failed to form m-SKPs whereas those from the murine vibrissae follicles did. Collectively, these findings reveal the potential for using expanded DF cultures to produce SKPs, the heterogeneity of SKP forming potential of skin from distinct anatomical locations and ages, and question the progenitor status of human hair follicle DP cells.

## Introduction

Human skin is an accessible source of progenitor cells termed skin derived precursors (SKPs) that were first isolated and differentiated along neural crest lineages in 2001 [1]. Although the multi-lineage potential of SKPs and their expression of multipotency markers has been well characterised [2]–[4] our understanding of factors influencing SKP formation and longevity remains limited.

SKPs are conventionally formed from dissociated dermis [1]–[4], alongside this stem cell capacity has been observed in monolayer fibroblast cultures [5]; it is therefore unsurprising that a recent study has described an alternative technique whereby SKP-like structures are formed from monolayer dermal cultures [6] (henceforth termed m-SKPs). However, many of the characteristics attributed to SKPs have yet to be described in these m-SKPs. In this study we investigated the multipotent marker expression and differentiation capacity of m-SKPs formed from adult dermal fibroblast (DF) cultures. Moreover, as SKPs formed directly from adult human skin lose expansion capacity and multipotency with increasing donor age [4] we also investigated the effects of monolayer passage number and cryopreservation on m-SKP yield, multipotency marker expression and differentiation capacity.

Neonatal foreskin has been identified as a particularly rich source of SKPs [2], however these tissues are obviously in short supply. Therefore, the question arises of whether there is an alternative/optimal adult body site for SKP isolation. It has previously been demonstrated that glabrous (smooth) and non-glabrous (hairy) skin differ in stem cell potential [3], we therefore quantified the m-SKP forming capacity of various monolayer DF cultures isolated from hair-dense and hair-sparse adult skin. A method of generating SKPs from freshly micro-dissected dissociated whole human hair follicle dermal papilla (DP) has also been described [7], leading some to suggest that the DP is the source of SKPs within the adult skin. We therefore also investigated m-SKP forming capacity of monolayer DP cultures.

In this study, we show that m-SKPs formed from healthy adult human DF retain multipotent characteristics associated with SKPs formed by traditional means. We also demonstrate that cryopreservation does not affect either m-SKP yield or character, whereas extensive monolayer passage does. Monolayer neonatal foreskin cultures at low passage yield significantly more m-SKPs than adult cultures, while within adult cultures hair dense regions yield significantly more m-SKPs than those established from hair sparse regions. After extensive monolayer passage, neonatal foreskin cultures did not display an enhanced m-SKP yield over adult hair dense cultures, but both yielded more than cultures from hair sparse regions. Interestingly, hair follicle DP cells cultured under identical conditions and from the same donor skin specimens as fibroblasts that readily formed m-SKPs did not form m-SKPs; whereas mouse vibrissae follicle DP monolayer cultures did.

The ability of adult DF to form m-SKPs which are morphologically and functionally similar to traditional SKPs, from monolayer cultures after multiple passage and cryopreservation highlights their potential for use in cell replacement studies and translational work. For the use of m-SKPs for more diagnostic or translational work we sought to determine whether the technique of forming m-SKPs from cryopreserved normal fibroblasts could be expanded to include fibroblasts isolated from patients with existing disorders. In this paper, we assessed the m-SKP forming potential of DF from Progeria patients, a disease that causes premature aging. Traditional monolayer progeria DF cultures display reduced proliferative ability; we found that these cells readily formed m-SKPs suggesting that this technique may be of use in increasing the yield and proliferative capability of traditionally difficult to culture diseased cells.

## Materials and Methods

### Isolation, Culture and Cryopreservation of Dermal Fibroblast and Hair Follicle Dermal Papilla Cultures

Project is covered under ethical guidelines from National Research Ethics Service (Reference: 05/MRE01/72). Normal human skin tissue was obtained from patients undergoing elective plastic surgeries, who provided written consent according to ethically approved guidelines, from Durham University Hospital (Durham, UK), The Royal Victoria Infirmary (Newcastle upon Tyne, UK) and the James Cook Hospital (Teesside, UK). Adult DF were isolated from adult dermal explants from both male and female donors ranging in age from 28 to 62 years from six hair sparse sites (breast, face and abdomen) and six hair rich sites (scalp, arm and abdomen). Papillary dermal tissue from the twelve adults and four neonatal foreskins (from separate individuals) were dissected under a stereo-dissecting microscope to approximately 3 mm×3 mm in size and cultured in fibroblast adherence media (Table S1A). Adherent cells were cultured in fibroblast proliferation media (Table S1A). Human and mouse DP were isolated as previously described [8], [9], with mouse DP coming from CD-1 strains. Established progeria DF cultures were a kind gift of Prof. C. J. Hutchison [10]. Cells were enzymatically passaged at ∼80% confluence using 0.25% Trypsin-EDTA (Invitrogen - 25200-072). Cells were cryopreserved (at a rate of −1°C/minute) in 90% foetal bovine serum (FBS - Sigma – F7524)/10% dimethyl sulfoxide (Sigma – D2650) in an isopropanol bath within a freezer maintained at a constant temperature of −140°C.

### Formation and Culture of m-SKPs

m-SKPs were formed, cultured and passaged using an adaption of the method previously described [3]. Briefly, monolayer cultures of varying passage number were all seeded at 25,000 cells/ml in SKP proliferation media (Table S1A). Cultures were agitated daily and supplemented with fresh 10× SKP proliferation media every four to five days. m-SKPs were passaged by dissociation with 1 mg/ml collagenase XI (Sigma – C9407) for 45 minutes before being re-seeded in a 1∶2 ratio, with 2× SKP proliferation media and the old conditioned media in a 1∶1 ratio.

### Immunofluorescent Analysis of Monolayer Cultures

Cells cultured as monolayers were fixed with 4% paraformaldehyde (PFA) for 1 hour, permeabilized with 0.1% Triton X-100 for 30 minutes and blocked with 3% bovine serum albumin (Sigma – A2153) for 1 hour at room temperature (RT). Samples were incubated with primary antibodies (Table S1B) overnight at 4°C before incubation with appropriate secondary antibodies (Table S1B) for 1 hour at RT, prior to counterstaining and mounting with VectaShield containing 4′,6-diamidino-2-phenylindole (DAPI – Vector Laboratories – H1500). Images were captured using Zeiss Imager M1 fluorescent microscope.

### Immunofluorescent Analysis of m-SKPs

m-SKPs were fixed in 4% PFA overnight at 4°C followed by incubation in 30% sucrose overnight at 4°C. m-SKPs were then snap frozen and embedded in optimal cutting temperature (OCT) compound (Fisher – LAMB/OCT) and sectioned at 10 µm. Sections were then stained and visualized as above.

### RNA Extraction and RT-PCR

RNA was extracted from cells using an RNeasy mini kit (Qiagen - 74104) and cDNA generated with Superscript III reverse transcriptase (Invitrogen - 18080-044) according to the manufacturer’s instructions. PCR was performed with *Taq* DNA polymerase (Invitrogen - 10966-018) using the primers and conditions described (Table S1C). All PCRs were performed alongside a negative control (without reverse transcriptase) and products were separated on a 2% agarose gel containing ethidium bromide with bands visualized under UV.

### Differentiation of m-SKPs

m-SKPs were dissociated using collagenase XI as described above. For adipogenic and osteogenic differentiation cells were seeded at 80,000 cells/35 mm dish and allowed to adhere overnight in SKP adherence media (Table S1A). Cells were then cultured in adipogenic and osteogenic differentiation media (Table S1A) for 14 days, with media changed every 3–4 days. Oil Red-O staining was used to detect lipids and Von Kossa staining to detect calcified deposits using methodology we have previously described [11].

For neuronal and Schwann cell differentiation cells were seeded at 25,000 cells/ml on laminin (0.02 mg/ml – Sigma - L4544) and poly-D-lysine (0.2 mg/ml – Sigma - P7280) coated glass coverslips and allowed to adhere overnight in SKP adherence media (as described above). Cells were then cultured in neuronal or Schwann cell differentiation media (Table S1A) for 28 days with media changed every 3–4 days. Immunofluorescent analysis (as described above) was used to assess S100β and β-III tubulin expression.

### Quantification of m-SKPs

m-SKPs were counted under a stereo-dissecting microscope under blind conditions. All data points are representative of three independent experiments and results are expressed as means±SEM. An ANOVA was used to compare data between control and test samples. Statistical significance was accepted at the P<0.05 level (*), P<0.01 level (**) and P<0.001 level (***).

## Results

### m-SKPs can be Routinely Formed and Passaged from Cryopreserved Human Dermal Fibroblasts

**Figure 1:**
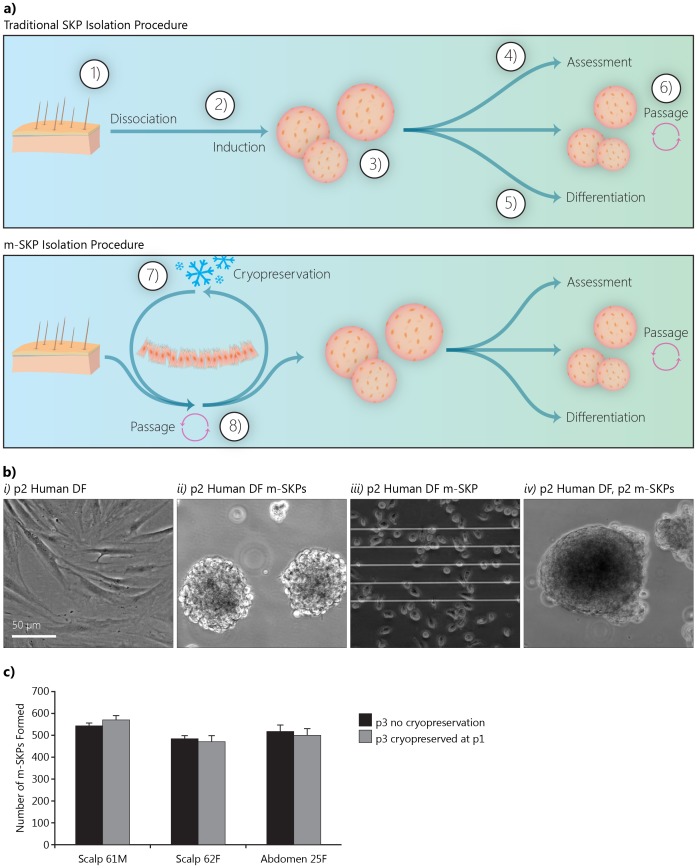
Monolayer cultured dermal fibroblasts yield m-SKPs after passage and cryopreservation. **a** ***i*** **)** Skin biopsies are dissociated *1)* and the resulting DF suspension is cultured in SKP proliferation media *2)*. SKPs are formed after approximately 7 days *3)*, at which point they can be assessed for marker expression *4)*, differentiated down neural and mesenchymal lineages *5)* or maintained *via* passage *6)*. **a** ***ii*** **)** DF isolated from skin biopsies are cultured as monolayers *7)* which can be passaged *8)* and cryopreserved *9*). These DF cultures form m-SKPs upon incubation with SKP proliferation media. **b)** Phase contrast images of **b** ***i*** **)** original monolayer normal adult human DF, **b** ***ii*** **)** m-SKPs derived from the DF culture in **b** ***i*** **), b** ***iii*** **)** collagenase XI treated m-SKPs (from **b** ***ii*** **)** in single cell suspension and **b** ***iv*** **)** passaged m-SKPs (derived from single cell suspension in **b** ***iii*** **)**. c) There was no difference in m-SKP yield from matched DF cultures expanded to p3, or those expanded to p3 after cryopreservation at p1. (DF – dermal fibroblasts, (m)-SKP – (monolayer) skin derived precursor).

m-SKPs formed using our isolation protocol (Figure 1A) from cultured adult DF at p3 and p12 were morphologically similar, with average diameters of 141.6±12.6 µm for p3 m-SKPs and 130.1±15.3 µm for p12 m-SKPs (data not shown). The first DF m-SKPs were identifiable after 7 to 11 days in SKP proliferation media and took on average 21 days to form. Furthermore, we found that m-SKPs derived from adult DF at p2 could be passaged at least twice (Figure 1B), and that cryopreservation of monolayer cultures at p1 did not affect m-SKP yield (Figure 1C).

### Nestin and Versican Expression is Up-regulated in Response to m-SKP Formation in Cryopreserved Human Adult Dermal Fibroblasts

**Figure 2:**
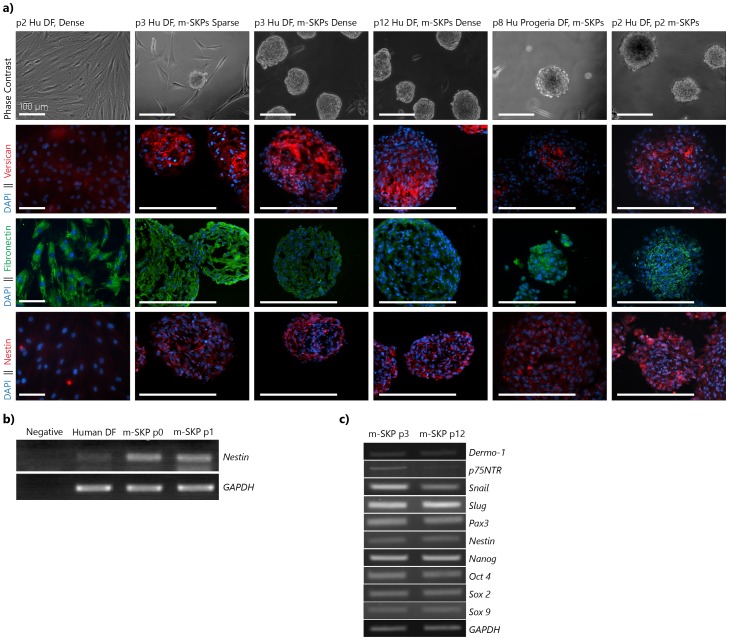
m-SKPs express markers associated with traditionally isolated SKPs. **a)** In response to m-SKP formation cryopreserved normal adult human DF from both low (p3 hair dense and hair sparse) and high (p12 hair dense) passage and cryopreserved diseased (acute progeria) human DF from intermediate passage (p8) up-regulate expression of nestin and versican protein. Moreover, nestin and versican expression are maintained in passaged m-SKPs (p2) derived from cryopreserved low passage normal adult human DF. DF express fibronectin which is maintained after m-SKP formation. **b)** Nestin mRNA expression is induced in p2 normal adult human DF upon m-SKP formation and maintained after passaging. **c)** m-SKPs formed from both low (p3) and high (p12) passage cryopreserved normal adult human DF express similar levels of *Dermo-1*, *Slug*, *Pax-3*, *Nestin*, *Nanog*, *Oct-4*, *Sox-2* and *Sox-9*. Levels of *p75NTR* and *Snail*, however, are reduced in m-SKPs formed from high (p12) passage cryopreserved normal adult human DF. (m-SKP – monolayer skin derived precursor, DF – dermal fibroblasts).

In monolayer culture adult human DF did not express the neural crest stem cell marker nestin or the undifferentiated mesenchymal stem cell marker versican. However, upon m-SKP formation both of these stem cell markers were up-regulated regardless of fibroblast passage number, body site or disease status (Figures 2A and 2B). Furthermore, neither nestin nor versican expression was altered upon subsequent passaging of these m-SKPs. In monolayer culture adult human DF expressed the mesenchymal stem cell-associated marker fibronectin (Figure 2A). Moreover, upon m-SKP formation and subsequent passaging fibronectin expression was unaltered in these cells (Figure 2A).

### m-SKPs Formed from p3 and p12 Cryopreserved Normal Human Adult Dermal Fibroblasts Isolated from Hair Dense Anatomical Regions have Similar Stem Cell Marker Expression Profiles

In order to compare m-SKPs with SKPs described in studies from dissociated tissues, we examined the expression of markers that have been well characterised in SKPs [2]. RT-PCR of six donors showed that m-SKPs from both p3 and p12 fibroblast cells of hair dense origin expressed transcripts for *Sox-2*, *Sox-9*, *Oct-4*, *Nanog*, *Nestin*, *Pax-3*, *Slug*, *Snail*, *Dermo-1* and *p75NTR* (Figure 2C). Moreover, *p75NTR* and *Snail* transcripts in m-SKPs derived from scalp fibroblast cultures were both reduced at p12 when compared to p3, while all other markers remained relatively constant with increasing passage number (Figure 2C) (percent reductions in transcript expression by densitometric analysis between p3 and p12 are summarised in Table 1).

**Table 1:** Densitometric analysis of percent decrease in *p75NTR* and *Snail* expression (p12 versus p3) in four populations of fibroblast m-SKPs.

Donor (age)	*p75NTR* percent reduction at p12	*Snail* percent reduction at p12
Abdomen female (25)	Not detected at either p3 or p12	40
Arm female (28)	Not detected at either p3 or p12	36
Scalp female (62)	64	44
Scalp male (61)	58	29

### m-SKPs Formed from Cryopreserved Normal Human Adult Dermal Fibroblasts can be Directed to Multiple Differentiation Pathways

#### Osteogenic

**Figure 3:**
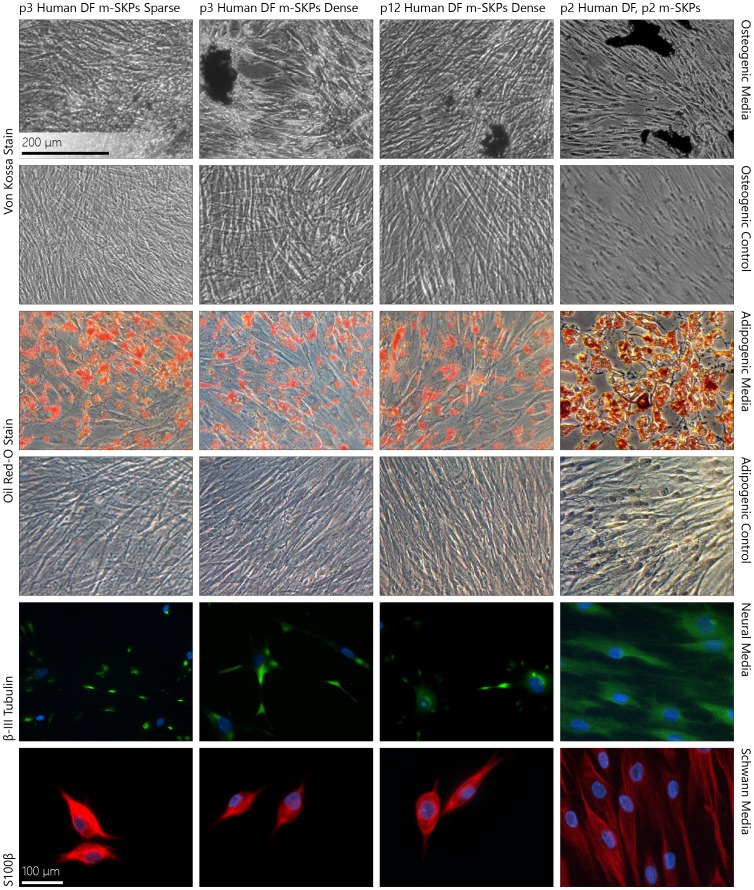
m-SKPs display multipotency which is preserved after m-SKP passage. m-SKPs derived from both low and high monolayer passage DF obtained from hair dense anatomical regions and low monolayer passage DF from hair sparse anatomical regions can be differentiated down osteogenic, adipogenic and neuronal lineages as shown by Von Kossa and Oil Red-O and β-III tubulin and S100β staining, respectively. Moreover this multipotency is preserved after two rounds of m-SKP passage. (m-SKP – monolayer skin derived precursor, DF – dermal fibroblasts).

After 14 days in osteogenic medium Von Kossa staining revealed discrete masses of calcification in cultures generated from m-SKPs produced from DF isolated from both hair rich (at p3 and p12) and hair sparse (at p3, as an insufficient number of m-SKPs was generated at p12) anatomical locations (Figure 3). Moreover, cultures generated from passaged m-SKPs produced from DF (p2) isolated from hair dense anatomical locations also produced calcified deposits (Figure 3). Negative controls cultured in minimal essential medium (MEM) supplemented with 10% FBS did not exhibit calcification (Figure 3).

#### Adipogenic

After 14 days in adipogenic medium Oil Red-O staining revealed lipid deposition in cultures generated from m-SKPs produced from DF isolated from both hair rich (at p3 and p12) and hair sparse (at p3) anatomical locations (Figure 3). Moreover, cultures generated from passaged m-SKPs (p2) produced from DF isolated from hair dense anatomical locations also revealed lipid deposition (Figure 3). Negative controls cultured in MEM supplemented with 10% FBS did not exhibit lipid deposition (Figure 3).

#### Neuronal and Schwann cell

After 28 days in neuronal or Schwann cell medium immunofluorescence staining revealed expression of the early neuronal marker β-III tubulin or the Schwann cell differentiation marker S100β, respectively, in cultures generated from m-SKPs produced from DF isolated from both hair rich (at p3 and p12) and hair sparse (at p3) anatomical locations (Figure 3). Moreover, cultures generated from passaged m-SKPs (p2) produced from DF isolated from hair dense anatomical locations also expressed these markers when in the corresponding medium for 28 days (Figure 3). Control cultures on coated glass slides in MEM supplemented with 10% FBS did not label positively for β-III tubulin or S100β (data not shown).

### High Levels of αSMA Expression Correlate with Enhanced m-SKP Formation in Cryopreserved Normal Human Adult Dermal Fibroblasts

**Figure 4:**
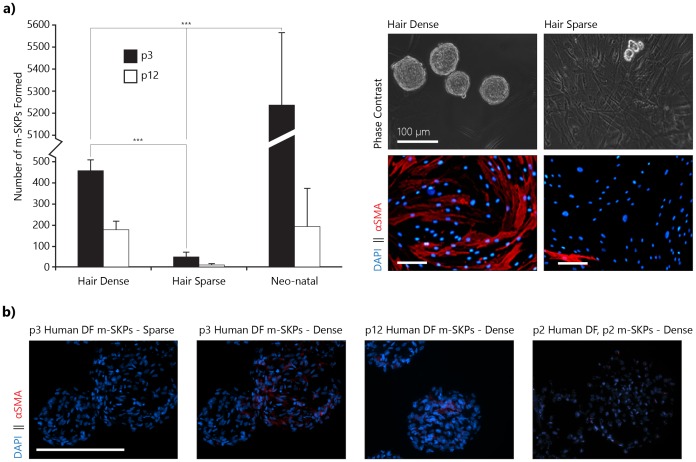
Adult hair dense biopsies yield more m-SKPs than hair sparse, m-SKP yield also correlates with increased αSMA expression in adult dermal fibroblasts. a) Normal adult human DF from hair dense anatomical regions form significantly more m-SKPs than DF from hair sparse anatomical regions (p<0.001, n = 6). However, neonatal dermal fibroblasts form significantly more m-SKPs than either (p<0.001, n = 4). Increasing passage number of monoculture fibroblasts reduces m-SKP forming capacity in all three groups, with the largest percentage reduction observed in neonatal cultures. In adult samples hair dense, high m-SKP yielding populations also display increased αSMA expression in monolayer culture. b) Upon formation of m-SKPs αSMA expression is lost in all samples. (m-SKP – monolayer skin derived precursor, DF – dermal fibroblasts).

A greater proportion of DF (p3) isolated from hair dense (scalp 84.2%±3.1, abdomen 76.5%±6.8 and arm 78.5%±5.5; *n* = 3) biopsies expressed αSMA than those isolated from hair sparse (breast 3.4%±1.2, face 2.9%±1.7 and neonate foreskin 3.4%±2.0; *n* = 3) biopsies (Figure 4A). Adult DF (p3) from hair dense biopsies with correspondingly high αSMA expression yielded more (p<0.001, n = *6*) m-SKPs than those from hair sparse biopsies. Neonatal foreskin DF (p3) cultures yielded significantly more (p<0.001, n = *4*) m-SKPs than either adult populations (Table 2). In all instances, m-SKP yield was strongly reduced at p12, with the largest percentage reduction observed in neonatal DF cultures (Figure 4A). m-SKPs from any source did not retain αSMA expression upon m-SKP formation (Figure 4B).

**Table 2:** m-SKP Yields from adult hair dense and hair sparse and neonatal monolayer cultures.

Donor site	Donor age/sex	*n = 3* average m-SKP count at p3	*n = 3* average m-SKP count at p12
**Adult hair dense**			
Scalp^†^	61/m	479	264
*Scalp^†^	62/f	481	245
Scalp	Unknown	234	78
Abdomen^†^	25/f	549	202
Arm	28/f	413	173
Abdomen^†^	64/f	583	174
**Adult hair sparse**			
Breast	47/f	177	29
Facial	Unknown	22	2
Abdomen	36/m	15	0
Breast	Unknown	23	2
Breast	28/f	32	6
*Facial	62/f	11	0
**Neonatal hair sparse**			
Foreskin	Neonatal	5880	118
Foreskin	Neonatal	4728	247
Foreskin	Neonatal	4462	162
Foreskin	Neonatal	5646	272

Counts of total m-SKPs formed from dermal fibroblast cells previously cultured as monolayers (*denotes m-SKPs formed from one specimen that contained both hair sparse and hair dense sites and ^†^denotes donors that formed fibroblast m-SKPs but did not form papilla m-SKPs. Counting was performed “blind”.

### Human Dermal Papilla do not Form m-SKPs

**Figure 5:**
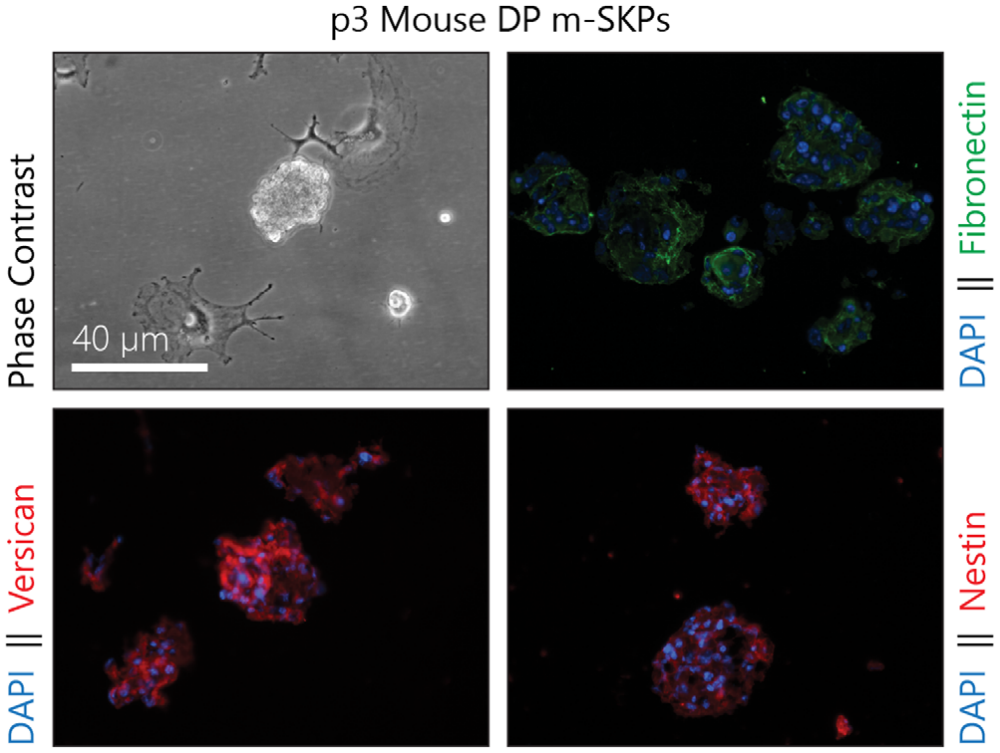
Mouse vibrissae, but not human follicle dermal papillae form m-SKPs. Normal adult human DP at p4 did not form m-SKPs, with cells remaining attached to the culture vessel surface. However, mouse DP at p3 formed small m-SKP cultures expressing versican, fibronectin and nestin. Mouse DP m-SKPs took longer to form and were smaller than human DF m-SKPs. (m-SKP – monolayer skin derived precursor, DP – dermal papilla).

The DP has been identified as a rich source of SKPs [7], is αSMA rich, fills a progenitor role and is obviously present more abundantly in hair dense regions [12]. Human DP were micro-dissected and cultured from biopsies before DF were isolated. These DP cultures were unable to form m-SKPs at p3, even those from biopsies that yielded large numbers of DF derived m-SKPs. Interestingly, mouse vibrissae follicle DP at p3 were capable of forming m-SKPs although they remained considerably smaller than human DF m-SKPs, and also took longer to form (Figure 5).

## Discussion

This study has shown that both normal and diseased adult DF and normal neonatal DF previously expanded in monolayer culture in conventional media are capable of forming m-SKPs. The method describing the formation of m-SKPs was recently reported using DF from young children [6], however, here were demonstrate that this method can be employed to establish m-SKPs from monolayer cultured, cryopreserved adult DF. Moreover, this capacity for m-SKP formation is unaffected by cryopreservation as expanding cells from explant initiation to p3 (with and without cryopreservation) did not affect the number of m-SKPs generated. This latter finding is relatively unsurprising as stem cells are routinely cryopreserved [13] for the treatment of many malignant and non-malignant diseases, a process that has been proven to be safe and not associated with significant adverse outcomes regarding failure to engraft, graft versus host disease, or engraftment failure [14].

With regard to stem cell markers, our findings were broadly similar to those previously described for SKPs [1]–[3]. In particular, our m-SKPs expressed neural crest, embryonic and mesenchymal stem cell markers. Additionally, like Gago *et al.* [4], we found p75NTR expression to be unique to scalp-skin (hair dense) derived SKPs. Moreover, Jinno *et al.* [15], also show that p75NTR expression is increased in adult rat skin compared to embryonic rat skin i.e. at ages reflecting hair dense and hair sparse anatomical regions, respectively. This finding may be explained by the fact that p75NTR protein expression exhibits significant hair cycle-dependent fluctuation, suggesting a possible role in human hair follicle biology [16]. We also observed decreased *p75NTR* and *snail* transcript expression with increasing passage number, suggesting prolonged monolayer culture is reducing progenitor cell population or capability. However, both high passage, low *p75NTR* and *snail* expressing cultures and hair sparse cultures with no *p75NTR* remained capable of differentiation down various lineages. As we were assaying total populations by PCR, rather than identifying localised p75NTR within specific cells, it may be that the reduced *p75NTR* expression was due to a reduction in progenitor cell population, rather than a population wide reduction resulting in a reduction in m-SKP yield. Interestingly Toma *et al.* described a similar reduction in *p75NTR* expression upon increasing SKP passage number [2].

Co-expression of versican, fibronectin and nestin is a widely used marker of mature SKPs. Monolayer cultures did not express versican or nestin but did express fibronectin; upon exposure to sphere forming conditions expression of versican and nestin was up-regulated in all m-SKPs derived from normal monolayer cultures, with no change in expression of fibronectin [1]–[3]. However, m-SKPs derived from patients with progeria, a disease where accelerated ageing is observed expressed less nestin and versican, suggesting that progeria m-SKPs, assayed at the same time-point as normal m-SKPs had not developed to the same degree. This could be accounted for due to the intrinsically slower proliferation in culture associated with these cells [10].

In line with previous observations with conventionally formed SKPs [1], [3], [17], m-SKPs derived from both hair dense and hair sparse sites showed adipogenic, osteogenic and neural differentiation capacity upon appropriate stimulation. We did not observe any site-specific differentiation capacity, and this capacity was maintained after m-SKP passage. The ability to use existing monolayer cultures or cryopreserved stocks to generate m-SKPs, which are morphologically and functionally identical to traditionally formed SKPs, demonstrates that they may be of use in cell replacement studies and translational work.

Questions about SKP origin and heterogeneity have featured prominently in previous studies in which researchers have observed differences in SKP formation derived from different donor sites. For example, SKPs were readily generated from neonatal foreskin [18] but some adult dermal sites have yielded relatively fewer [19].

Our observations for m-SKPs mirror those described for SKPs, in that neonatal foreskin-derived fibroblasts at p3 showed the highest m-SKP forming potential of the cell types investigated, by a factor of around 10 compared to adult tissue. Within adult cultures, there was no discernible trend linking m-SKP forming potential and donor age; however, there were trends relating to donor site and monolayer passage number. That is cells derived originally from non-glabrous skin produced greater numbers of m-SKPs compared to glabrous skin, and low passage fibroblast cultures produced more m-SKPs than high passaged ones. Intriguingly, although m-SKPs derived from cultured foreskin cells formed significantly more m-SKPs than both adult sources at p3, at p12 there was no significant difference between the numbers of m-SKPs formed by foreskin cultures, or hair dense DF cultures. These results may link with our previous discussion about *p75NTR* expression, with the possibility that after increasing age/extended culture there is a reduction in an m-SKP forming progenitor cell population as suggested by Gago *el al.* [4], or that as a whole the population is less capable of forming m-SKPs.

Within adult dermal tissue we found that hair dense regions yield more m-SKPs than hair sparse, results similar to those previously described by Biernaskie *et al.* [3], suggesting that the hair follicle may contain an enriched SKP forming niche. Indeed, Hunt *et al.* [7], have gone further and proposed the human DP as an enriched source of SKPs. Interestingly and in line with previous reports [20] we have found that enriched α-SMA expression within hair dense cultures, over hair sparse equivalents, correlated with an increased m-SKP forming potential. This may be due to α-SMA positive cells being more capable of focal adhesion during m-SKP formation. However, upon formation of m-SKPs α-SMA expression was lost, perhaps because other adhesion molecules play a greater role in maintaining m-SKP integrity or more simply by the omission of FBS from SKP proliferation media [21].

The possibility of a straightforward correlation between the levels of α-SMA expression in the cultured dermal cells and their m-SKP forming capacity was undermined by our finding that cultured human hair follicle DP, that strongly express α-SMA, consistently did not yield m-SKPs. We currently have no explanation as to why human hair follicle DP cells did not form m-SKPs, while cultured fibroblasts from the same tissue specimens, or freshly extracted DP were capable of doing so. Kawase [22] found that supplementing SKPs proliferation media with transforming growth factor beta (TGF-β), increased SKP formation. It may be possible that factors secreted by skin fibroblasts (but not cultured DP cells) enhance m-SKP formation.

Other evidence points to the distinctive character of papilla derived SKPs. For example papilla-derived SKPs themselves express α-SMA [7] but in the current study none of the fibroblast derived m-SKPs expressed α-SMA. Moreover, transcripts of the *Oct-4* pluripotency gene have been identified in porcine back skin SKPs [23], murine SKPs [24] and in human fibroblast m-SKPs produced in the current work. Yet *Oct-4* was not found in the specialised SKPs derived from hair follicle DP cells [7]. This combination of undetectable *Oct-4* and detectable α-SMA suggests that specialised and intricate differences could exist between dermal fibroblast and papilla derived SKPs. When human DP cells are grown in monolayer culture they undergo a rapid loss of papilla specific identity [25], perhaps this also impacts on their capacity to become m-SKPs? Interestingly, it has been shown that human DP, which strongly express versican *in vivo* maintain this expression when cultured as spherical hanging drops, but lose it when cultured as monolayers [26].

Taken together our findings show that it is possible to generate m-SKPs from cryopreserved healthy and diseased monolayer cultures which are morphologically and functionally similar to traditionally isolated SKPs. Whilst neonatal sources yield the greatest number of m-SKPs at early passage, cultures from adult hair dense regions yield reasonable m-SKP numbers, and this capacity is maintained to a greater degree over extended passage. These findings will be of use to the field by greatly increasing the cell populations available for SKP research.

## Supporting Information

Table S1 **Media, antibody and primer details. a)** Composition of fibroblast adherence media, fibroblast proliferation media, SKP adherence media, SKP proliferation media, adipogenic differentiation media, osteogenic differentiation media, neuronal differentiation media and Schwann differentiation media. **b**) Table of primary and secondary antibodies used in immunofluorescent studies. **c**) Table of primers used in PCR reactions. Conditions for PCR were as follows: 95°C for 4 minutes followed by 35 cycles of 95°C for 30 seconds, gene specific annealing temperature for 30 seconds and 72°C for 40 seconds with a final extension of 72°C for 7 minutes.
(DOCX)

## References

[1] Toma JG, Akhavan M, Fernandes KJ, Barnabé-Heider F, Sadikot A, et al (2001) Isolation of multipotent adult stem cells from the dermis of mammalian skin. Nature cell biology 3: 778–784. 11533656 10.1038/ncb0901-778

[2] Toma JG, McKenzie IA, Bagli D, Miller FD (2005) Isolation and characterization of multipotent skin-derived precursors from human skin. Stem cells 23: 727–737. 15917469 10.1634/stemcells.2004-0134

[3] Biernaskie JA, McKenzie IA, Toma JG, Miller FD (2006) Isolation of skin-derived precursors (SKPs) and differentiation and enrichment of their Schwann cell progeny. Nature protocols 1: 2803–2812. 17406538 10.1038/nprot.2006.422

[4] Gago N, Pérez-López V, Sanz-Jaka JP, Cormenzana P, Eizaguirre I, et al (2009) Age-dependent depletion of human skin-derived progenitor cells. Stem cells 27: 1164–1172. 19418448 10.1002/stem.27

[5] Lorenz K, Sicker M, Schmelzer E, Rupf T, Salvetter J, et al (2008) Multilineage differentiation potential of human dermal skin-derived fibroblasts. Experimental dermatology 17: 925–932. 18557932 10.1111/j.1600-0625.2008.00724.x

[6] Wenzel V, Roedl D, Gabriel D, Gordon LB, Herlyn M, et al (2012) Naive adult stem cells from patients with Hutchinson-Gilford progeria syndrome express low levels of progerin in vivo. Biology Open 1: 516–526. 23213444 10.1242/bio.20121149 PMC3509444

[7] Hunt DPJ, Morris PN, Sterling J, Anderson JA, Joannides A, et al (2008) A highly enriched niche of precursor cells with neuronal and glial potential within the hair follicle dermal papilla of adult skin. Stem cells 26: 163–172. 17901404 10.1634/stemcells.2007-0281

[8] Jahoda CA, Oliver RF (1984) Vibrissa dermal papilla cell aggregative behaviour in vivo and in vitro. Journal of embryology and experimental morphology 79: 211–224. 6716045

[9] Messenger AG (1984) The culture of dermal papilla cells from human hair follicles. The British journal of dermatology 110: 685–689. 6375713 10.1111/j.1365-2133.1984.tb04705.x

[10] Richards SA, Muter J, Ritchie P, Lattanzi G, Hutchison CJ (2011) The accumulation of un-repairable DNA damage in laminopathy progeria fibroblasts is caused by ROS generation and is prevented by treatment with N-acetyl cysteine. Human molecular genetics 20: 3997–4004. 21807766 10.1093/hmg/ddr327

[11] Jahoda CAB, Whitehouse J, Reynolds AJ, Hole N (2003) Hair follicle dermal cells differentiate into adipogenic and osteogenic lineages. Experimental dermatology 12: 849–859. 14714566 10.1111/j.0906-6705.2003.00161.x

[12] Driskell RR, Clavel C, Rendl M, Watt FM (2011) Hair follicle dermal papilla cells at a glance. Journal of cell science 124: 1179–1182 doi:10.1242/jcs.082446. 21444748 10.1242/jcs.082446 PMC3115771

[13] Baharvand H, Salekdeh GH, Taei A, Mollamohammadi S (2010) An efficient and easy-to-use cryopreservation protocol for human ES and iPS cells. Nature protocols 5: 588–594. 20203673 10.1038/nprot.2009.247

[14] Stockschläder M, Hassan HT, Krog C, Krüger W, Löliger C, et al (1997) Long-term follow-up of leukaemia patients after related cryopreserved allogeneic bone marrow transplantation. British journal of haematology 96: 382–386. 9029030 10.1046/j.1365-2141.1997.d01-2032.x

[15] Jinno H, Morozova O, Jones KL, Biernaskie JA, Paris M, et al (2010) Convergent genesis of an adult neural crest-like dermal stem cell from distinct developmental origins. Stem cells (Dayton, Ohio) 28: 2027–2040. 10.1002/stem.525 PMC3087810 20848654

[16] Adly MA, Assaf HA, Hussein MRA (2009) Expression pattern of p75 neurotrophin receptor protein in human scalp skin and hair follicles: Hair cycle-dependent expression. Journal of the American Academy of Dermatology 60: 99–109. 19103362 10.1016/j.jaad.2008.09.060

[17] Lavoie J-F, Biernaskie JA, Chen Y, Bagli D, Alman B, et al (2009) Skin-derived precursors differentiate into skeletogenic cell types and contribute to bone repair. Stem cells and development 18: 893–906. 18834279 10.1089/scd.2008.0260

[18] Fernandes KJL, Kobayashi NR, Gallagher CJ, Barnabé-Heider F, Aumont A, et al (2006) Analysis of the neurogenic potential of multipotent skin-derived precursors. Experimental neurology 201: 32–48. 16678161 10.1016/j.expneurol.2006.03.018

[19] Joannides A, Gaughwin P, Schwiening C, Majed H, Sterling J, et al (2004) Efficient generation of neural precursors from adult human skin: astrocytes promote neurogenesis from skin-derived stem cells. Lancet 364: 172–178. 15246730 10.1016/S0140-6736(04)16630-0

[20] Jahoda CA, Reynolds AJ, Chaponnier C, Forester JC, Gabbiani G (1991) Smooth muscle alpha-actin is a marker for hair follicle dermis in vivo and in vitro. Journal of cell science 99 (Pt 3): 627–636. 10.1242/jcs.99.3.627 1939373

[21] Steinbach SK, El-Mounayri O, DaCosta RS, Frontini MJ, Nong Z, et al (2011) Directed differentiation of skin-derived precursors into functional vascular smooth muscle cells. Arteriosclerosis, thrombosis, and vascular biology 31: 2938–2948. 10.1161/ATVBAHA.111.232975 21852558

[22] Kawase Y, Yanagi Y, Takato T, Fujimoto M, Okochi H (2004) Characterization of multipotent adult stem cells from the skin: transforming growth factor-beta (TGF-beta) facilitates cell growth. Experimental cell research 295: 194–203. 15051502 10.1016/j.yexcr.2003.12.027

[23] Dyce PW, Zhu H, Craig J, Li J (2004) Stem cells with multilineage potential derived from porcine skin. Biochemical and biophysical research communications 316: 651–658. 15033449 10.1016/j.bbrc.2004.02.093

[24] Guo W, Miao C, Liu S, Qiu Z, Li J, et al (2009) Efficient differentiation of insulin-producing cells from skin-derived stem cells. Cell proliferation 42: 49–62. 19143763 10.1111/j.1365-2184.2008.00573.x PMC6496823

[25] Ohyama M, Kobayashi T, Sasaki T, Shimizu A, Amagai M (2012) Restoration of the intrinsic properties of human dermal papilla in vitro. Journal of cell science. doi:10.1242/jcs.105700. 10.1242/jcs.105700 22623722

[26] Higgins CA, Richardson GD, Ferdinando D, Westgate GE, Jahoda CAB (2010) Modelling the hair follicle dermal papilla using spheroid cell cultures. Experimental dermatology 19: 546–548 doi:10.1111/j.1600-0625.2009.01007.x. 20456497 10.1111/j.1600-0625.2009.01007.x

