# Supplementary material for: Generation and Characterization of Multipotent Stem Cells from Established Dermal Cultures
Source: PLoS One. 2012 Nov 30;7(11):e50742. doi: 10.1371/journal.pone.0050742 (PMC3511366; doi:10.1371/journal.pone.0050742)
Supplement: Table S1 — Media, antibody and primer details. a) Composition of fibroblast adherence media, fibroblast proliferation media, SKP adherence media, SKP proliferation media, adipogenic differentiation media, osteogenic differentiation media, neuronal differentiation media and Schwann differentiation media. b) Table of primary and secondary antibodies used in immunofluorescent studies. c) Table of primers used in PCR reactions. Conditions for PCR were as follows: 95°C for 4 minutes followed by 35 cycles of 95°C for 30 seconds, gene specific annealing temperature for 30 seconds and 72°C for 40 seconds with a final extension of 72°C for 7 minutes. (DOCX) [file pone.0050742.s001.docx]

**Table S1 a)**

| **Reagent** | **Concentration** | **Supplier** | **Catalogue Number** |
| --- | --- | --- | --- |
| **Fibroblast Adherence Media – MEM – 42360-081** | | | |
| Foetal Bovine Serum (FBS) | 20% | Sigma | F7524 |
| Amphotericin B | 0.5 µg/ml | Invitrogen | 15290018 |
| Penicillin/Streptomycin (Pen/Strep) | 100 units/100 µg/ml | Invitrogen | 15070063 |
| **Fibroblast Proliferation Media - MEM** | | | |
| FBS | 10% | Sigma | F7524 |
| Amphotericin B | 0.5 µg/ml | Invitrogen | 15290018 |
| Pen/Strep | 100 units/100 µg/ml | Invitrogen | 15070063 |
| **1x SKP Proliferation Media – DMEM:F12 (3:1) – Invitrogen - 10567-014 & 31765-035** | | | |
| Epidermal Growth Factor (EGF) | 20 ng/ml | Peprotech | AF-100-15 |
| Basic Fibroblast Growth Factor (bFGF) | 40 ng/ml | Peprotech | 100-18B |
| B27 | 2% v/v | Invitrogen | 17504044 |
| Amphotericin B | 0.5 µg/ml | Invitrogen | 15290018 |
| Pen/Strep | 100 units/100 µg/ml | Invitrogen | 15070063 |
| **SKP Adherence Media – DMEM:F12 (3:1)** | | | |
| EGF | 20 ng/ml | Peprotech | AF-100-15 |
| bFGF | 40 ng/ml | Peprotech | 100-18B |
| B27 | 2% v/v | Invitrogen | 17504044 |
| FBS | 5% v/v | Sigma | F7524 |
| Amphotericin B | 0.5 µg/ml | Invitrogen | 15290018 |
| Pen/Strep | 100 units/100 µg/ml | Invitrogen | 15070063 |
| **Adipogenic Differentiation Media - MEM** | | | |
| 3-isobutyl-1-methylxanthine (IBMX) | 0.45 nM | Sigma | I7018 |
| Insulin | 2.07 µM | Sigma | I3536 |
| Dexamethasone | 100 nM | Sigma | D4902 |
| Rabbit Serum | 15% v/v | Sigma | R4505 |
| Amphotericin B | 0.5 µg/ml | Invitrogen | 15290018 |
| Pen/Strep | 100 units/100 µg/ml | Invitrogen | 15070063 |
| **Osteogenic Differentiation Media - MEM** | | | |
| Dexamethasone | 100 nM | Sigma | D4902 |
| β-glycerophosphate | 10 mM | Sigma | G9422 |
| L-Ascorbic acid 2-phosphate | 50 µM | Sigma | A8960 |
| FBS | 10% v/v | Sigma | F7524 |
| Amphotericin B | 0.5 µg/ml | Invitrogen | 15290018 |
| Pen/Strep | 100 units/100 µg/ml | Invitrogen | 15070063 |
| **Schwann Cell Differentiation Media - DMEM:F12 (3:1)** | | | |
| Forskolin | 5 µM | Sigma | F3917 |
| Heregulin-1β | 50 ng/ml | Peprotech | 100-03 |
| N_2_ Supplement | 2 % v/v | Invitrogen | 17502 |
| FBS | 1 % v/v | Sigma | F7524 |
| **Neural Cell Differentiation Media - DMEM:F12 (3:1)** | | | |
| Neurotrophiin-3 | 10 ng/ml | Peprotech | 450-03 |
| Nerve Growth Factor | 50 ng/ml | Peprotech | 450-01 |
| Brain-Derived Neurotrophic Factor | 50 ng/ml | Peprotech | 450-02 |
| FBS | 1 % v/v | Sigma | F7524 |

**Table S1 b)**

| **Primary Antibodies** | | | | |
| --- | --- | --- | --- | --- |
| **Target** | **Company** | **Catalogue Number** | **Diluent** | **Dilution** |
| **Versican** | DSHB | 12C5 | 3% BSA | 1:10 |
| **Fibronectin** | Sigma | F3648 | 3% BSA | 1:100 |
| **Nestin** | Millipore | MAB5326 | 3% BSA | 1:100 |
| **β-III Tubulin** | Sigma | T8660 | 3% BSA | 1:100 |
| **S100β** | Sigma | S2532 | 3% BSA | 1:100 |
| **αSMA** | MUbio | MUB0100S | 3% BSA | 1:100 |
| **Secondary Antibodies** | | | | |
| **Mouse** | Invitrogen | A21203 | 3% BSA | 1:500 |
| **Rabbit** | Invitrogen | A21206 | 3% BSA | 1:500 |

**Table S1 c)**

| **Target RNA** | **Primer Sequence (5’-3’)** | | **Annealing Temp** |
| --- | --- | --- | --- |
|  | **Forward** | **Reverse** |  |
| ***Dermo-1*** | AAGGGGGAGGTAAAATTGAAA | CTAAACTAAATTACTAAATAATTATC | 52^0^C |
| ***P75NTR*** | TGGGCCCAGAAGGTTGCGATGAA | AAAGGGGCCCCAGAACCAAACACA | 60^0^C |
| ***Snail*** | TGGCCTGTCTGCGTGGGTTTTTGT | CCTGGGCTCGGGGCATCTCA | 60^0^C |
| ***Slug*** | CATCTTTGGGGCGAGTGAGTCC | CCCCCGTGTGAGTTCTAATGTGTC | 60^0^C |
| ***Pax-3*** | CATCCGGCCCTGCGTCATCTC | TGGCCTTCTTCTCGCTTTCCTCTG | 60^0^C |
| ***Nestin*** | GGCTCCAAGACTTCCCTCAG | TAAGAAAGGCTGGCACAGGT | 60^0^C |
| ***Nanog*** | CAGAAGGCCTCAGCACCTAC | GCCTCCAAGTCACTGGCAG | 60^0^C |
| ***Oct-4*** | CGAAAGAGAAAGCGAACCAC | GTGAAGTGAGGGCTCCCATA | 60^0^C |
| ***Sox-2*** | ACCAGCTCGCAGACCTACAT | ATGTGTGAGAGGGGCAGTGT | 60^0^C |
| ***Sox-9*** | GCTCTGGAGACTTCTGAACGA | AAGTCGATAGGGGGCTGTCT | 60^0^C |
| ***GAPDH*** | CGGAGTCAACGGATTTGGTCG | AGCCTTCTCCATGGTGGTGAA | 58^0^C |
